# Clinical Usefulness of Urinary Fatty Acid Binding Proteins in Assessing the Severity and Predicting Treatment Response of Pneumonia in Critically Ill Patients: A Cross-Sectional Study

**DOI:** 10.1097/MD.0000000000003682

**Published:** 2016-05-13

**Authors:** Tsung-Cheng Tsao, Han-Chen Tsai, Shi-Chuan Chang

**Affiliations:** From the Institute of Emergency and Critical Care Medicine, National Yang-Ming University (T-CT, S-CC) and Department of Nursing (T-CT, H-CT) and Department of Chest Medicine (S-CC), Taipei Veterans General Hospital, Taipei, Taiwan.

## Abstract

To investigate the clinical relevance of urinary fatty acid binding proteins (FABPs), including intestinal-FABP, adipocyte-FABP, liver-FABP, and heart-FABP in pneumonia patients required admission to respiratory intensive care unit (RICU).

Consecutive pneumonia patients who admitted to RICU from September 2013 to October 2014 were enrolled except for those with pneumonia for more than 24 h before admission to RICU. Pneumonia patients were further divided into with and without septic shock subgroups. Twelve patients without infection were enrolled to serve as control group. Urine samples were collected on days 1 and 7 after admission to RICU for measuring FABPs and inflammatory cytokines. Clinical and laboratory data were collected and compared between pneumonia and control groups, and between the pneumonia patients with and without septic shock.

There were no significant differences in urinary levels of various FABPs and inflammatory cytokines measured on day 1 between control and pneumonia groups. Urinary values of intestine-FABP (*P* = 0.020), adipocyte-FABP (*P* = 0.005), heart-FABP (*P* = 0.025), and interleukin-6 (*P* = 0.019) were significantly higher and arterial oxygen tension/fraction of inspired oxygen (PaO_2_/FiO_2_, P/F) ratio (*P* = 0.024) was significantly lower in pneumonia patients with septic shock on day 1 than in those without septic shock. After multivariate analysis, adipocyte-FABP was the independent factor (*P* = 0.026). Urinary levels of FABPs measured on day 7 of pneumonia patients were significantly lower in the improved than in nonimproved groups (*P* = 0.030 for intestine-FABP, *P* = 0.003 for adipocyte-FABP, *P* = 0.010 for heart-FABP, and *P* = 0.008 for liver-FABP, respectively). After multivariate analysis, adipocyte-FABP was the independent factor (*P* = 0.023).

For pneumonia patients required admission to RICU, urinary levels of adipocyte-FABP on days 1 and 7 after admission to RICU may be valuable in assessing the pneumonia severity and in predicting treatment response, respectively. Further studies with larger populations are needed to verify these issues.

## INTRODUCTION

In Europe, lungs were the most common site of infection, and accounted for 64% of all infections in the European Prevalence of Infection in intensive Care Study.^1^ Mortality rates remain high in patients with severe pneumonia despite advances in antimicrobial therapy.^2–5^ Ultimate mortality rates of pneumonia patients requiring admission to intensive care unit (ICU) were reported to be ≥40%.^6,7^

A simple and reliable method that can assess the severity of pneumonia may potentially improve the triage or initial management of the patients by the physicians to determine whether close observation or aggressive treatment is more appropriate than conservative management. Correct and early identification of severe community-acquired pneumonia cases will reduce the inappropriate use of intravenous antibiotics.^8^ However, accurately assessing the severity of pneumonia remains to be a major clinical challenge. Certain score systems for determining the severity of pneumonia develop including pneumonia severity index and CURB-65 (confusion, urea nitrogen, respiratory rate, blood pressure, age > 65 years)^9,10^; however, these score systems may be not suitable for the pneumonia patients requiring admission to ICU.

Previous studies revealed that serum and urinary levels of fatty acid binding proteins (FABPs) might provide information about the specific tissue damage. The liver-FABP, intestine-FABP, adipose-FABP, and heart-FABP can be measured in extracellular fluids including plasma and urine.^11–14^ Interleukin-8 (IL-8), a proinflammatory cytokine, and chemokine, could be detected in the urine of patients with urinary tract infection.^15^ Some inflammatory cytokines could be detected and appeared to be higher in pleural fluid and bronchoalveolar lavage fluid in infectious diseases.^16,17^ Taken together, we hypothesized that these biomarkers described as above might be present in the urine of pneumonia patients and could be used to reflect the severity of pneumonia and to predict outcome of such patients.

In this study, we intended to measure urine levels of several types of FABPs and certain inflammatory cytokines in pneumonia patients requiring admission to respiratory ICU (RICU) and explored their clinical relevance.

## PATIENTS AND METHODS

### Study Design

This single-center, prospective observation study intended to investigate the clinical importance of certain FABPs, some inflammatory cytokines, and biomarkers in the urine. The study was approved by the Institute Review Board of Taipei Veterans General hospital (VGHIRB No. 2013-04-030BC), and written informed consent of each patient was obtained from the patients or their authorized representatives before entering the study.

### Patient Selection

**FIGURE 1:**
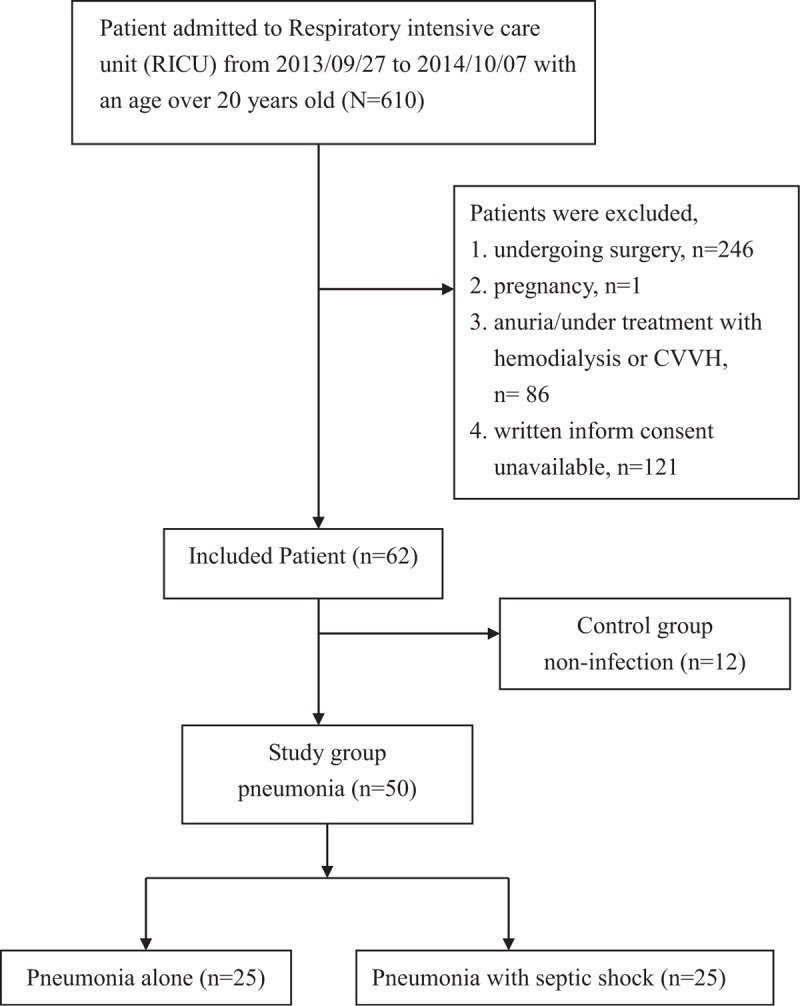
Flow diagram of patients in the control and study groups.

From September 2013 to October 2014, consecutive patients with an age ≥ 20 years who were admitted to a 24-bed RICU at Taipei Veterans General Hospital were eligible for this study. The patients who had symptoms and signs of lower respiratory tract infection and pneumonia shown on chest radiograms and/or computed tomography of the chest were included this study. The excluding criteria were as follows: patients had pneumonia for more than 24 h before admission to RICU; patients undergoing thoracic surgery, pregnancy; patients without urine and required hemodialysis or continuous venovenous hemofiltration; patients without indwelling urinary catheter; and patients without providing written inform consent. Finally, a total of 50 pneumonia patients were enrolled into this study and 12 patients who admitted to RICU without clinical evidence of any infection and inflammation were enrolled to serve as control group (Figure 1).

### Collection of Urine and Measurement of FABPs and Cytokines

The 10-mL urine samples were collected in the early mornings of days 1 and 7 after admission to RICU. The cell-free supernatants of urine sample were stored at −70°C after centrifuge until assayed. The commercial available enzyme-linked immunosorbent assay kits were used to measure urine levels of IL-1β, IL-6, IL-8, and IL-10 (R&D System, Minneapolis, MN) and to measure urine levels of intestine-FABP, adipose-FABP, heart-FABP, and liver-FABP (Hycult biotech Inc., Plymouth Meeting, PA).

### Data Collection

The demographic and clinical data were collected from the medical record after the patients admitted to RICU including clinical features and laboratory data like body temperature, mentality, respiratory rate, heart rate, mean arterial pressure, arterial blood gas analysis, arterial oxygen tension (PaO_2_)/fraction of inspired oxygen (FiO_2_) ratio (PaO_2_/FiO_2_ ratio, P/F ratio), arterial oxygen saturation (SPO_2_), presence of pleural effusion, hemoglobin, leukocytes, blood urea nitrogen, creatinine, glucose, sodium, potassium, C-reactive protein (CRP), procalcitonin, and acute physiology and chronic health evaluation II (APACHE II) score. The studied subjects were determined by the study period from September 2013 to October 2014 approved by the institutional review board of our institute.

### Definition of Clinical Improvement

On the day 7 after admission to RICU, the pneumonia patients were classified into improved and nonimproved groups based on the criterion as follows: chest radiograms, clinical features including fever, leukocytes, mentality change, gross appearance of respiratory secretions, newly developed respiratory symptoms and signs, oxygenation expressed as P/F ratio, and persistence or additional use of inotropic agents.^18^ The patients who had at least 1 of the followings: failure to improve the P/F ratio, persistence of fever (≥38°C) or hypothermia (<35.5°C) plus purulent respiratory secretions, worsening of the pulmonary infiltrates by >50%, persistence or additional use of inotropic agents, and occurrence of multiple organ dysfunction were classified into nonimproved group.^19^ The improved pneumonia patients had an improvement of pulmonary infiltrates as evidenced by reducing the pneumonia lesion >20% and/or increased visualization of lung markings within the pneumonia lesions on chest radiograms, an improvement of P/F ratio for >50 mm Hg in the setting of FiO_2_ ≤ 40% and none of the criteria mentioned in the nonimproved group.

### Statistical Analysis

Most data were not normally distributed. The continuous variables were expressed as median with interquartile range. Statistical comparisons between 2 groups were performed by using Mann–Whitney *U* test. Comparisons of categorical variables between the 2 groups were made by using the chi-squared test and/or Fisher exact test, when appropriate. All statistical tests were 2-tailed. The independent variables in assessing the severity of pneumonia and in predicting treatment response were assessed by stepwise logistic regression analysis. Statistical significance was defined as *P* < 0.05. Statistical analysis was performed using SPSS version 18.0 (SPSS, Chicago, IL).

## RESULTS

### Patients Enrolled

A total of 50 pneumonia patients were enrolled for this study. Twelve patients who had no pneumonia, any infection, and/or inflammatory diseases were enrolled as control group. Of those with noninfectious or noninflammatory diseases, heart failure was found in 9 patients, and carbon monoxide intoxication, benzodiazepine intoxication, and cancer with brain metastasis were found in 1 patient each.

### Comparisons Between Pneumonia Patients and Noninfectious Patients

Comparisons of demographic characteristics, urine levels of FABPs and other biomarkers on day 1 after admission to RICU are shown in Table 1. There were no significant differences in all data measured except for that APACHE II scores were significantly higher in noninfection patients than in pneumonia patients.

**TABLE 1:**
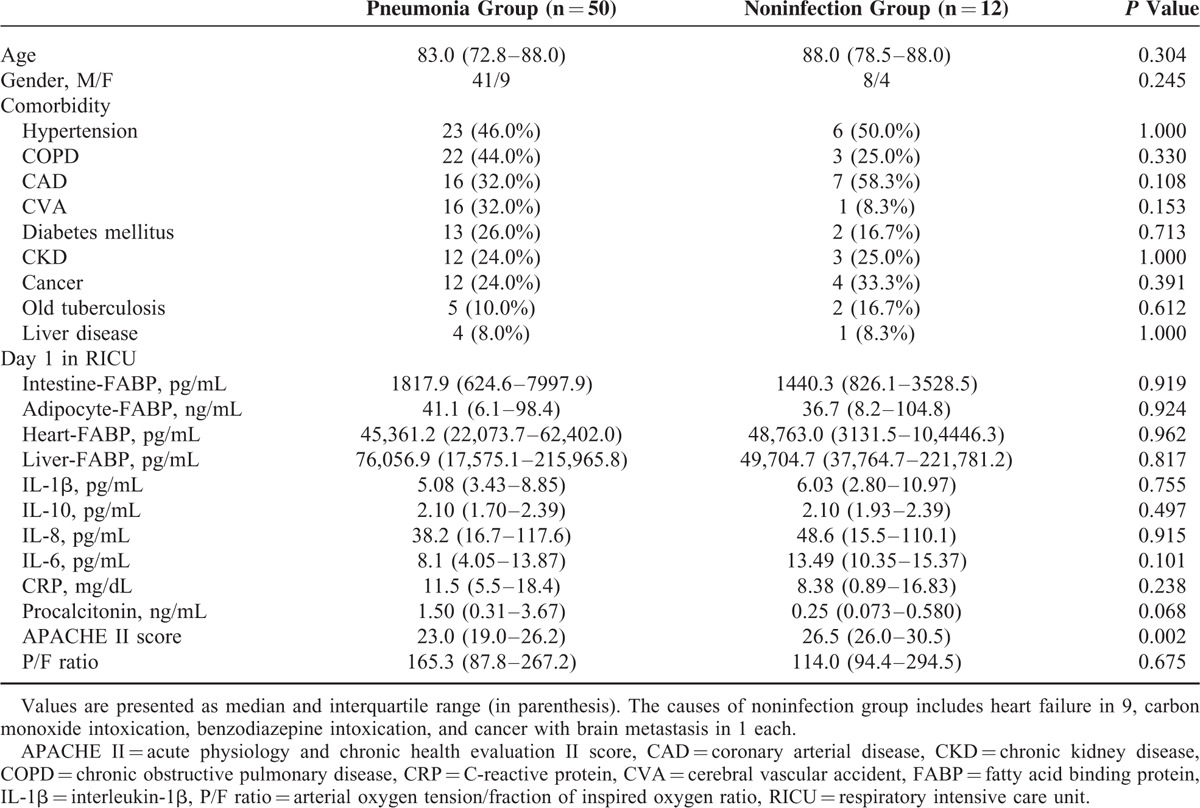
Comparisons Between Pneumonia and Noninfection Groups

### Comparisons of Urinary Biomarkers Between Pneumonia Patients With and Without Septic Shock

The pneumonia patients were divided into the patients with and without septic shock groups. There were 25 patients in each group, respectively. The data on days 1 and 7 after admission to RICU between the 2 groups were compared. The results showed that urinary values of intestine-FABP, adipocyte-FABP, heart-FABP, and IL-6 were significantly higher and P/F ratio was significantly lower in pneumonia with septic shock than in those without septic shock on day 1 after RICU admission (Table 2). Of these, adipocyte-FABP was the independent factor after stepwise logistic regression analysis. However, there were no significant differences in liver-FABP, IL-1β, IL-10, IL-8, CRP, procalcitonin, and APACHE II scores between the 2 groups. On day 7 after RICU admission, the values of adipocyte-FABP and APACHE II score were significantly lower in pneumonia patients than in those with septic shock (Table 2).

**TABLE 2:**
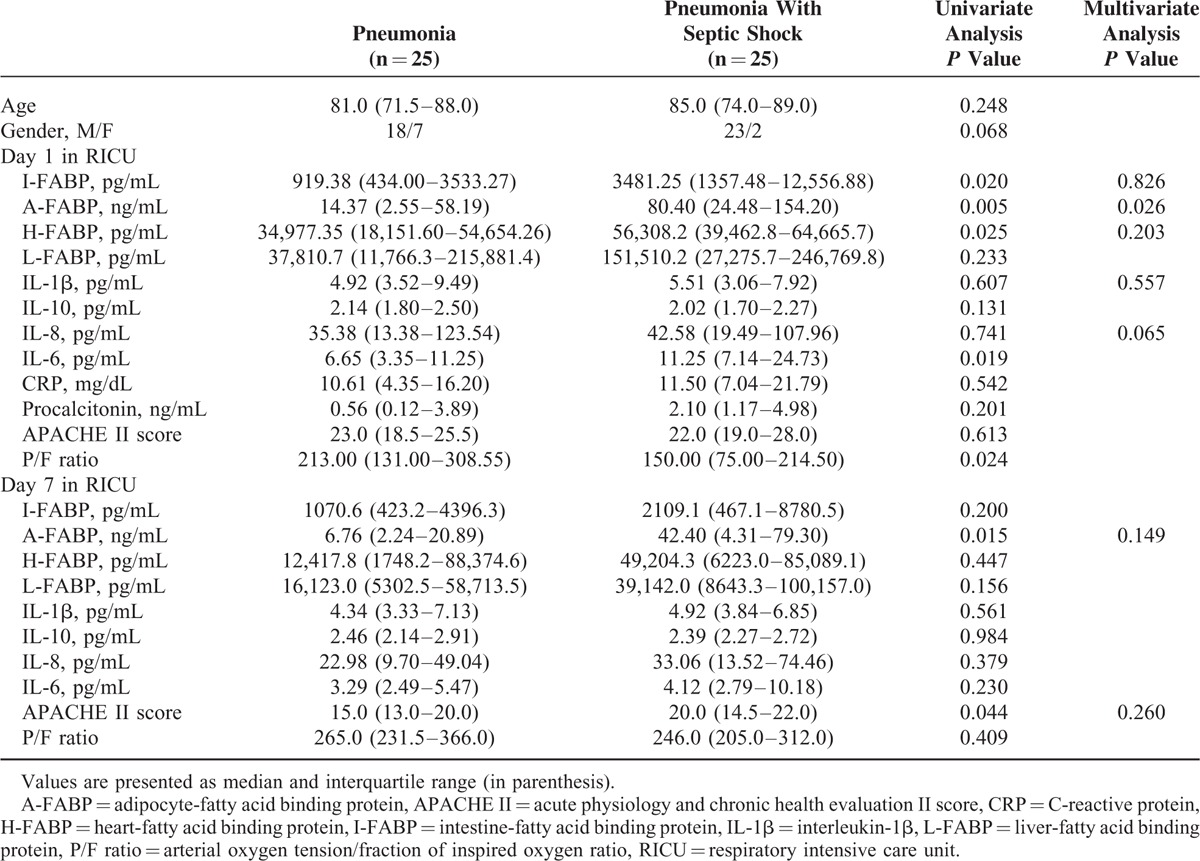
Comparisons Between Pneumonia Patients With and Without Septic Shock

### Comparisons Between Pneumonia Patients With and Without Clinical Improvement

We divided pneumonia patients into improved and nonimproved groups based on the treatment response as evidenced by clinical features, serial chest radiograms, hemodynamics, and oxygenation index (P/F ratio). There were 32 and 18 patients in improved and nonimproved groups, respectively. Comparisons of the data between the 2 groups are shown in Table 3. The data on day 1 after admission to RICU showed no significant differences except for APACHE II score were significantly higher in improved than in nonimproved groups. The values of urinary intestine-FABP, adipocyte-FABP, heart-FABP, and liver-FABP on day 7 after RICU admission were significantly lower in improved than in nonimproved groups. Of these, adipocyte-FABP was the independent factor after stepwise logistic regression analysis.

**TABLE 3:**
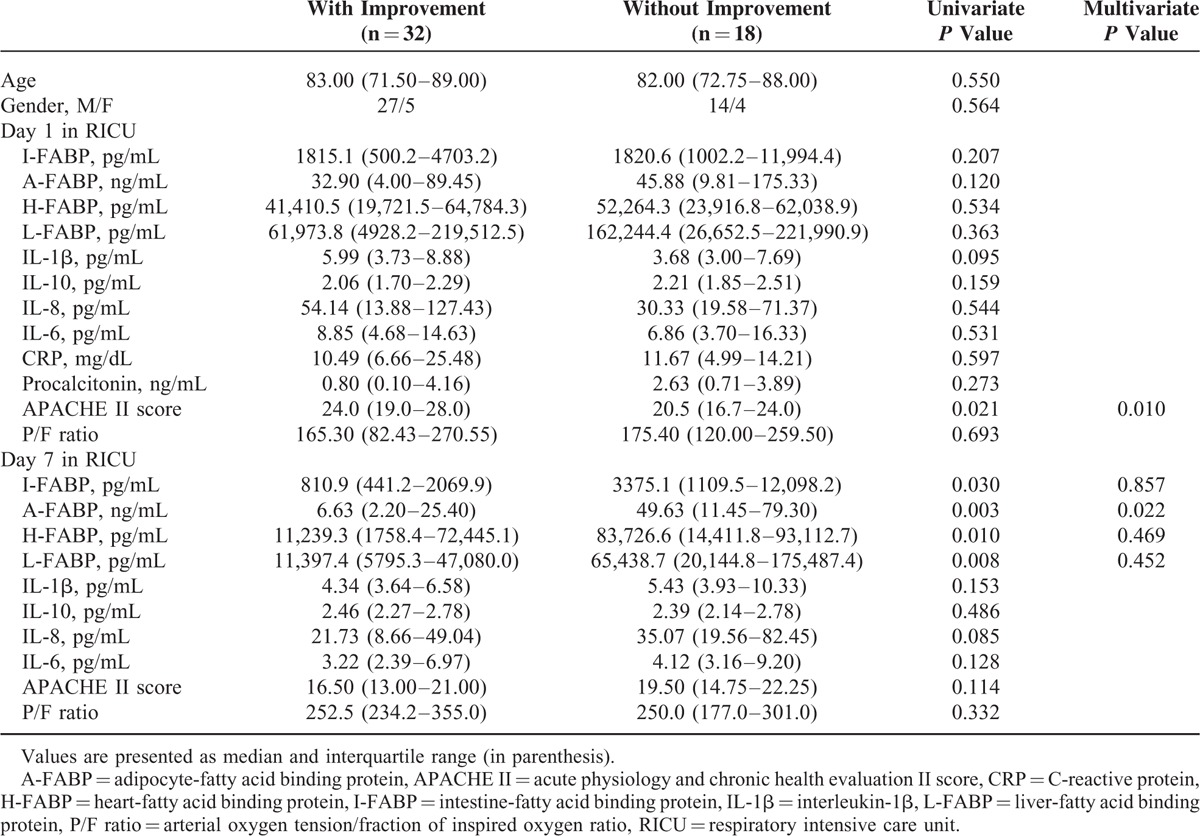
Comparisons Between Pneumonia Patients With and Without Improvement

## DISCUSSION

This is the first study to investigate the clinical relevance of urinary FABPs in pneumonia patients required admission to ICU, to the best of our knowledge. The results indicated that urinary levels of adipocyte-FABP could be of value in assessing the severity of pneumonia in such patients. In addition, urinary levels of adipocyte-FABP could be of value in predicting treatment response of pneumonia in such patients. Other FABPs and inflammatory cytokines in the urine measured in this study failed to provide any benefits in these issues.

FABPs, a group of small intracellular proteins, are abundantly expressed in almost all tissues that bind reversely with fatty acid and other lipids.^20^ FABPs were named based on the tissues they were discovered or found to be dominant in the specific tissues. However, this nomenclature is misleading because several types of FABPs can be found in more than 1 tissue. In normal condition, FABPs cannot be detected in the peripheral blood or urine. However, in various pathologic conditions, some types of FABPs could be detected in peripheral blood and urine.^13,14,21,22^ FABPs can bind a variety of long-chain fatty acids and retinoic acid, and are involved in the regulation of important biologic processes, including inflammation, lipid glucose homeostasis, and cell proliferation and differentiation. Furthermore, plasma levels of various FABPs may serve as biomarkers of specific tissue injury including heart-FABP for acute and chronic heart diseases, combination of heart-FABP and liver-FABP for kidney disorders, intestine-FABP for intestinal disorders, brain-type FABP and heart-FABP for brain disorders, respectively.^23^

No significant differences in urinary levels of various FABPs and inflammatory cytokines measured were found between pneumonia and control (noninfection and noninflammatory) groups. The findings highly suggested that urinary levels of FABPs and inflammatory cytokines could not be used to differentiate between infection and noninfection conditions in critically ill patients required admission to ICU (Table 1). Further studies with larger populations are needed to verify these issues.

The studied on the clinical relevance of adipocyte-FABP in respiratory diseases have been limited, to our knowledge. Adipocyte-FABP was initially identified in the adipocytes. Subsequently, adipocyte-FABP was found in macrophages and was found to be involved in the inflammation. Some studies reported that adipocyte-FABP might be involved in the allergic airway inflammation and bronchopulmonary dysplasia.^24,25^ Adipocyte-FABP was detected in bronchial microvasculature of the patients with bronchopulmonary dysplasia and in bronchial epithelial cells in patients with allergic airway inflammation, respectively. Furthermore, adipocyte-FABP appears to play a role in the pathogenesis of these disorders. Lungs have numerous macrophages in their alveoli, endothelial cells in bronchial microvasculature and broad alveolar capillary network, and epithelial cells in their bronchial trees. Accordingly, increased urinary levels of adipocyte-FABP in pneumonia patients can be expected.

The clinical relevance of various FABPs in pneumonia patients required admission to RICU has not yet been investigated. The present study indicated that the urinary levels of adipocyte-FABP on day 1 after admission to RICU were significantly higher in pneumonia patients with septic shock than in those without septic shock. Furthermore, the urinary levels of adipocyte-FABP on day 7 after admission to RICU were significantly lower in pneumonia patients with clinical improvement than in those without clinical improvement. Taken together, urinary levels of adipocyte-FABP can be of considerable value in assessing the severity of pneumonia in the patients required admission to RICU and in predicting the treatment response in such patients.

Previous studies indicated that blood levels of heart-FABP might serve as a prognostic factor for 28-day mortality in patients with severe sepsis and septic shock, and that plasma levels of heart-FABP were higher in patients with multiorgan dysfunction syndrome caused by respiratory infection than in healthy subjects.^26,27^ In agreement with previous studies, the present study indicated that urinary levels of heart-FABP were significantly higher in the patients with septic shock than in those without septic shock. However, our results failed to show that urinary levels of heart-FABP could predict the treatment response and serve as a prognostic marker for pneumonia patients required admission to ICU. The discrepancy between our and previous studies may be explain in part by pooling sepsis patients with different underlying causes into analyses in the previous studies.

One previous study indicated that serum levels of adipocyte-FABP were associated with insulin resistance and could predict the outcome of critically ill patients.^28^ The results of the study indicated that blood levels of adipocyte-FABP might be of value in predicting outcome of critically ill patients; however, the study pooled the patients with critical illnesses caused by a variety of underlying disorders. The results of the study indicated that sepsis patients had higher values of serum adipocyte-FABP than did nonsepsis patients, and serum adipocyte-FABP was an independent factor for worse hospital survival. The results of the previous study may support the results of our study that urinary levels of adipocyte-FABP was the independent factor for assessing the severity of pneumonia in patients required admission to RICU and for poor treatment response of such patients. Further prospective studies with large populations are needed to verify these issues.

There are some limitations of the present study. First, we did not measure the serum or blood levels of various FABPs and inflammatory cytokines. It is uncertain that urinary levels of various FABPs and inflammatory cytokines in pneumonia patients may surrogate those in the blood. Renal dysfunction may affect the urinary levels of FABPs and inflammatory cytokines although significant renal dysfunction was not found in our patients. It is unclear whether plasma levels of FABPs and inflammatory cytokines in pneumonia patients will be affected, including secretion or degradation, by the kidneys and/or liver. Previous study indicated that both urinary and plasma levels of intestine-FABP might be of value in early diagnosis of intestine ischemia.^14^ Accordingly, it is very simple and easy to collect urine samples in the context of similar clinical relevance of urinary and blood levels of adipocyte-FABP. Second, the number of cases studied is limited. Because the patient selection criteria of the present study were relatively stringent, the patients who did not meet the selection criteria were excluded. Further studies with larger populations are needed to elucidate the results of the present study. Third, the number of the patients in control group was limited in the present study, and most patients in control group had cardiac failure or tissue hypoxia caused by carbon monoxide intoxication. It is plausible that cardiac dysfunction and/or tissue hypoxia may affect and induce subsequent elevation of blood levels of various FABPs. Although urinary levels of various FABPs and inflammatory cytokines showed no significant difference between pneumonia and control groups, urinary levels of adipocyte-FABP appeared to be of value in assessing the severity of pneumonia and predicting the outcome of pneumonia patients required admission to RICU.

In summary, our results suggested that urinary levels of adipocyte-FABP might serve as a new biomarker in assessing the severity of pneumonia and in predicting the outcome of pneumonia in critically ill patients who required admission to ICU.
